# The value of CT-based radiomics in predicting the prognosis of acute pancreatitis

**DOI:** 10.3389/fmed.2023.1289295

**Published:** 2023-11-29

**Authors:** Ming Xue, Shuai Lin, Dexuan Xie, Hongzhen Wang, Qi Gao, Lei Zou, Xigang Xiao, Yulin Jia

**Affiliations:** ^1^Department of Radiology, First Affiliated Hospital of Harbin Medical University, Harbin, China; ^2^Department of Magnetic Resonance, First Affiliated Hospital of Harbin Medical University, Harbin, China

**Keywords:** acute pancreatitis, computed tomography, modified CT severity index, radiomics, prognosis

## Abstract

**Purpose:**

Early judgment of the progress of acute pancreatitis (AP) and timely intervention are crucial to the prognosis of patients. The purpose of this study was to investigate the application value of CT-based radiomics of pancreatic parenchyma in predicting the prognosis of early AP.

**Materials and methods:**

This retrospective study enrolled 137 patients diagnosed with AP (95 cases in the progressive group and 42 cases in the non-progressive group) who underwent CT scans. Patients were randomly divided into a training set (*n* = 95) and a validation set (*n* = 42) in a ratio of 7: 3. The region of interest (ROI) was outlined along the inner edge of the pancreatic parenchyma manually, and the Modified CT Severity Index (MCTSI) was assessed. After resampling and normalizing the CT image, a total of 2,264 radiomics features were extracted from the ROI. The radiomics features were downscaled and filtered using minimum redundancy maximum correlation (mRMR) and the least absolute shrinkage and selection operator algorithm (LASSO) regression, in turn, and the more optimal subset of radiomics features was selected. In addition, the radiomics score (rad-score) was calculated for each patient by the LASSO method. Clinical data were also analyzed to predict the prognosis of AP. Three prediction models, including clinical model, radiomics model, and combined clinical–radiomics model, are constructed. The effectiveness of each model was evaluated using receiver operating characteristic (ROC) curve analysis. The DeLong test was employed to compare the differences between the ROC curves. The decision curve analysis (DCA) is used to assess the net benefit of the model.

**Results:**

The mRMR algorithm and LASSO regression were used to select 13 radiomics features with high values. The rad-score of each texture feature was calculated to fuse MCTSI to establish the radiomics model, and both the clinical model and clinical–radiomics model were established. The clinical–radiomics model showed the best performance, the AUC and 95% confidence interval, accuracy, sensitivity, and specificity of the clinical–radiomics model in the training set were 0.984 (0.964–1.000), 0.947, 0.955, and 0.931, respectively. In the validation set, they were 0.942 (0.870–1.000), 0.929, 0.966, and 0.846, respectively. The Delong test showed that the predictive efficacy of the clinical–radiomics model was higher than that of the clinical model (*Z* = 2.767, *p* = 0.005) and the radiomics model (*Z* = 2.033, *p* = 0.042) in the validation set. Decision curve analysis demonstrated higher net clinical benefit for the clinical–radiomics model.

**Conclusion:**

The pancreatic parenchymal CT clinical–radiomics model has high diagnostic efficacy in predicting the progression of early AP patients, which is significantly better than the clinical or radiomics model. The combined model can help identify and determine the progression trend of patients with AP and improve the prognosis and survival of patients as early as possible.

## Introduction

Acute pancreatitis (AP) is an inflammatory disease of the exocrine pancreas with a complex and variable clinical course. It is a common acute abdomen of the digestive system. The pathogenesis is due to the abnormal activation of pancreatic enzymes that destroy the pancreas itself and surrounding tissues and organs, with the local inflammatory infiltration of the pancreas as the main feature. The etiology of AP is diverse, mainly biliary tract diseases, alcoholism, hypertriglyceridemia, and less commonly drugs, endoscopic retrograde cholangiopancreatography, postoperative period, metabolic factors, infections, heredity, autoimmune disorders, and trauma (1). According to the pathology, AP is classified into interstitial edematous pancreatitis (EEP) and acute necrotizing pancreatitis (ANP) (2).

Patients can feel severe abdominal pain and often trigger systemic inflammatory response syndrome (SIRS) and multiple organ dysfunction syndrome (MODS) and other severe complications. Then, this triggers pancreatic necrosis and persistent organ failure, which in severe cases can even lead to death with a mortality rate of 1–5% (3). Numerous studies have shown that controlling inflammation within 72 h of onset is crucial for reducing the incidence of complications and mortality in patients (4, 5).

Therefore, early prediction of the development of acute pancreatitis and taking reasonable measures timely are essential for the prognosis of patients. The occurrence of complications can be used as a valid indicator for prognostic assessment of pancreatitis (6). Complications include both immediate and long-term complications. Immediate complications include bleeding, pancreatic leakage, and gastrointestinal perforation (7). However, long-term complications are symptoms that lead to long-term weakness, disease recurrence, and endocrine and exocrine pancreatic insufficiency in some cases.

Clinicians diagnose AP mainly by observing patients’ symptoms, signs, and changes in laboratory indicators. However, early clinical symptoms and signs of AP are not specific. Serum amylase and lipase levels do not fully reflect the severity and progression of AP (8, 9). CRP > 150 mg/L suggests a complex course of acute pancreatitis. It has a sensitivity of 85%, but it is not specific. Procalcitonin (PCT) is also considered as a marker to evaluate the prognosis of acute pancreatitis. It is more responsive in the acute phase and can respond to bacterial and/or fungal infections or sepsis (10). Several scoring systems are now clinically available to assess the severity and prognosis of AP, such as the Bedside Index for Severity in Acute Pancreatitis (BISAP) and MCTSI (11). BISAP can identify patients early with a high risk of complications and death, including five indicators: BUN, impaired mental status, SIRS, age, and pleural effusion (12). The MCTSI has a high value in predicting severe acute pancreatitis, pancreatic necrosis, and organ failure (13). They have similar predictive efficacy for AP severity (14, 15). Except for the MCTSI system, all clinical scoring systems are based on clinical information and laboratory data. Although they reflect the pathologic and physiologic status of the patient, they may overlook both the pathoanatomical changes and local complications of AP. Imaging diagnosis is the basis for accurate clinical treatment, and CT is the main method to assess AP complications (16). In the early stage of AP, some patients’ pancreatic parenchymal changes are not significant on CT plain scan. Radiomics is the more novel technology of the moment. It applies high-throughput computation to rapidly extract features from tomograms and quantify them. It converts digital medical images into high-dimensional data with the aim of revealing biomedical images that reflect underlying pathophysiological information that cannot be observed by the naked eye through quantitative image analysis (17). A large number of studies have confirmed that a single radiomics model has great value in predicting the severity and recurrence of acute pancreatitis (18, 19). However, there is currently no research exploring the value of clinical–radiomics models in predicting the prognosis of AP patients.

This study is based on pancreatic CT plain scan images for radiomics analysis and fusion of clinical data. Exploring the prediction of complications in pancreatitis based on clinical–radiomics models to determine the prognosis of early AP.

## Materials and methods

### Patients

A total of 137 patients from April 2021 to November 2022 at the First Affiliated Hospital of Harbin Medical University were retrospectively collected, and imaging data and relevant clinical laboratory data of patients with a clinical diagnosis of AP were enrolled. Ethics committee approval was granted for this retrospective study, and the requirement for written informed consent was waived. The diagnostic criteria of AP were based on the 2012 revised Atlanta Classification of AP (2).

Inclusion criteria: ① Patients with the first onset of pancreatitis; ② the time interval between the onset and the examination was not more than 1 week; ③ good CT imaging quality.

Exclusion criteria: ① Patients with incomplete images, poor quality, or incomplete patient case information data; ② autoimmune pancreatitis, trauma, or recurrent pancreatitis and AP due to pancreatic tumor; ③ difficulty in outlining pancreatic parenchymal ROI.

Patients were divided into progressive and non-progressive groups according to the presence or absence of new local or systemic complications or exacerbation of complications. Patients were randomly divided into a training set and a validation set in a ratio of 7:3.

### Clinical information

The medical records were reviewed to collect baseline clinical and imaging information, including sex, age, BISAP, CPR, PCT, and MCTSI. The BISAP and MCTSI criteria are shown in Tables 1, 2.

**Table 1:** Bedside index of severity of acute pancreatitis (BISAP).

Risk factors	Scoring criteria	Scores
BUN	>25 mg/dL	1
	≤25 mg/dL	0
Impaired mental status	2	1
	1	0
SIRS	≥2 Criteria	1
	<2 Criteria	0
Age	>60 years	1
	≤60 years	0
Pleural effusion	Presence	1
	Absence	0

**Table 2:** MCTSI scores.

Index		MCTSI
Pancreatic inflammation	Normal pancreas	0
	Intrinsic pancreatic abnormalities with or without inflammatory changes in peripancreatic fat	2
	Pancreatic or peripancreatic fluid collection or peripancreatic fat necrosis	4
Pancreatic necrosis	None	0
	30% or less	2
	More than 30%	4
Extrapancreatic complications	one or more of pleural effusion, ascites, vascular complications, parenchymal complications, and/or gastrointestinal involvement	2

BISAP is an abbreviation for five indicators. They are as follows: B: BUN; I: impairment; S: SIRS; A: age; P: pleural effusion. Note: SIRS has two or more of the following signs: ① temperature > 38°C or < 36°C; ② heart rate > 90 beats/min; ③ respiration >20 breaths/min or PaCO_2_ < 32 mmHg; ④ white blood cell count >12.0 × $10^{9}$/L or < 4.0 × $10^{9}$/L or infantile cells >10%.

### CT image acquisition and image analysis

Siemens 64-row spiral CT was used to scan the mid-abdomen of the AP patient, with the patient in the supine position and hands raised flat over the head. Scanning with advanced head. Image acquisition parameters: tube voltage of 120 kV, tube current of 220 mA, layer thickness of 5 mm, interlayer of 5 mm, DFOV of 32 cm × 32 cm, rotation speed of 0.28 s/turn, pitch of 1.7, image layer thickness of 5 mm, matrix 512 × 512, and reconstructed thin layer of 1-mm image.

**Figure 1:**
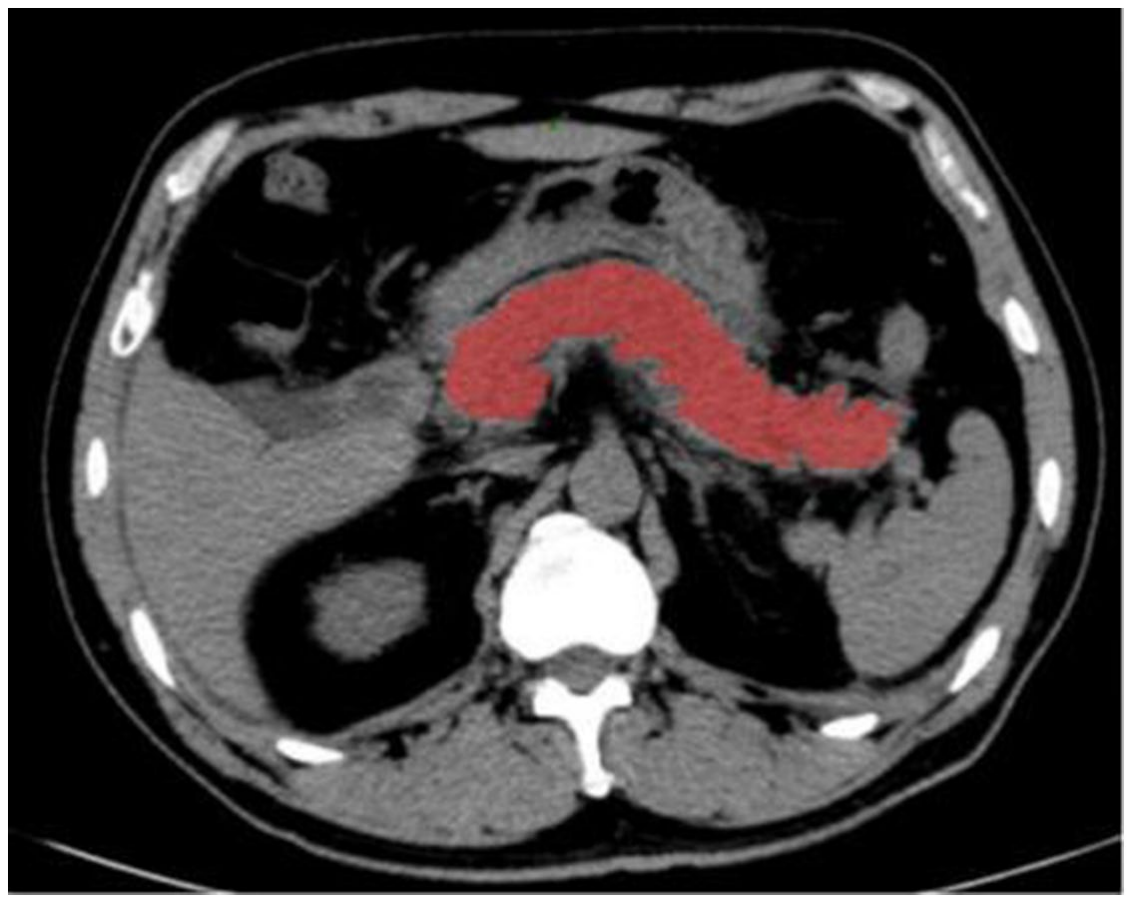
Manually delineate the ROI of pancreatic parenchyma with the uAI Research Portal.

The CT plain scan images of all patients were exported in DICOM format from the image archiving and communication system PACS workstation, and all of them were uploaded to the uAI Research Portal (uAI Research Portal version: 20220915, Shanghai United Imaging Intelligence, Co., Ltd.). Moreover, the radiomics feature extraction, feature selection, and machine learning models building were established on the uAI Research Portal (version: 20220915, Shanghai United Imaging Intelligence, Co., Ltd.), which was integrated with PyRadiomics (version 2.2.0), Scikit-Learn (version 1.2.0), and so on. The CT images were analyzed by two radiologists with 10 and 20 years of clinical experience blinded to outline the region of interest (ROI) along the edge of the pancreatic parenchyma by manual segmentation (Figure 1), and MCTSI scoring was performed, and in case of disagreement, the decision was made after discussion between the two physicians.

### Image preprocessing and feature extraction

To ensure repeatability, gray intensity normalization and resampling were performed to eliminate the heterogeneity between different scanners before feature extraction. Images were resampled to 1 mm × 1 mm × 1 mm voxels using the B-Spline interpolation method and normalized by subtracting the window level (WL: 30) and dividing by the window width (WW: 300). The extractable feature groups include first-order features, shape, and texture features (gray-level co-occurrence matrix [GLCM], gray-level travel length matrix [GLRLM], gray-level size zone matrix [GLSZM], gray-level dependence matrix [GLDM], and neighborhood gray-tone difference matrix [NGTDM]). The filter transforms include 14 filters, such as the Laplace-Gaussian filter, wavelet analysis, and local binary mode transform. A total of 2,264 radiomic features were finally extracted from the pancreatic parenchyma.

Intraclass correction coefficients (ICCs) were used to calculate intra-observer and inter-observer reproducibility (20). Two radiologists, A and B, manually delineate ROIs for all patient images. Moreover, 2 weeks later, radiologist B performed a second manual delineation to select image features with inter-observer and intra-observer ICCs >0.75.

### Filtering and establishment of radiomics labels

To avoid overfitting, it is necessary to reduce the dimensionality of the image data before the establishment of the radiomics label. The obtained radiomic features were reduced and filtered by using mRMR algorithm and LASSO regression to obtain the optimal subset of features, and the rad-score of each patient was calculated for each patient based on feature weights.

### Construction of the radiomics model

The obtained rad-score was fused with the MCTSI score to create a radiomics model. The baseline data were used to build a clinical model. A joint clinical–radiomics model was established by combining the two. The AUC values of the three models were analyzed using ROC curves. Compare the performance of different models in the training and validation sets. The performance of the same model is also compared in the training and validation sets. The DeLong test was used to compare the statistical differences among the three models. Evaluate clinical–radiomics characteristics by univariate and multivariate logistic regression analyses. The net clinical benefit of the models was compared by DCA.

### Statistical analysis

Statistical analysis was performed using SPSS (version 25.0, IBM) and R statistical software (Version 4.1.0). Clinical data for the training set and validation set were analyzed according to the variable type. Continuous variables that obeyed normal distribution are presented as mean ± standard deviation and analyzed by *t*-test for comparison of differences between groups; continuous variables that did not obey normal distribution are presented as median (interquartile range, IQR), and Wilcoxon rank sum test was used for comparison of differences between groups. Categorical variables were presented as frequency, and differences between groups were compared using the chi-square test or Fisher’s exact test for comparison of differences between groups. *p* < 0.05 was considered statistically different. The AUC and 95% confidence interval, accuracy, sensitivity, and specificity were used to evaluate the performance of the models, and the DeLong test was used to compare the differences between the ROC curves of the three models. DCA is used to compare net clinical benefit.

## Results

### Clinical characteristics and MCTSI scores

A total of 137 AP patients were enrolled in this study, which consisted of 84 male and 53 female cases with a mean age of 43 (35, 53) years. Patients were divided into progressive (*n* = 95) and non-progressive (*n* = 42) groups. There were 95 cases in the training set (66 cases in the progressive group and 29 cases in the non-progressive group) and 42 cases in the validation set (29 cases in the progressive group and 13 cases in the non-progressive group).

In both the training set and validation set, the BISAP (*Z* = −5.19; –3.55, *P* < 0.001) and PCT (*Z* = –3.92; –2.2, *P* < 0.001; *p* = 0.028) in the progression group were higher than in the non-progression group, while the gender differences were not statistically significant (*Z* = 2.27; 0.14, *p* = 0.707; 0.132). In the training set, the age in the progressive group was younger than the non-progressive group (*Z* = 2.11, *p* = 0.035), and the CRP was higher than the non-progressive group (*Z* = –3.05, *p* = 0.002) (Table 3).

**Table 3:** Intergroup comparison of clinical indicators and MCTSI Scores in the training set and validation set for patients in the progressive and non-progressive groups.

Project	Training set (*n* = 95)		χ^2^/z	*P*	Validation set (*n* = 42)		χ^2^/z	*P*
	Non-progressive group (*n* = 29)	Progressive group (*n* = 66)			Non-progressive group (*n* = 13)	Progressive group (*n* = 29)		
Gender			2.27	0.1322			0.14	0.7066
Male	21 (72.41)	37 (56.06)			7 (53.85)	19 (65.52)		
Female	8 (27.59)	29 (43.94)			6 (46.15)	10 (34.48)		
Age	47 (40.00,54.00)	39 (32.00,50.75)	2.11	0.0348	45.62 ± 16.83	43.52 ± 15.39	0.40	0.6935
BISAP	1 (1,1)	2 (2,3)	–5.19	<0.0001	1 (0,1)	2 (1,3)	–3.55	0.0004
CRP	141 (94.4,191.0)	201 (141.8,289.6)	–3.05	0.0023	185 (34,250)	220.58 (161,392)	−1.80	0.0725
PCT	0.79 (0.46,1.50)	1.93 (0.91,4.24)	−3.92	0.0001	1.1 (0.25,1.60)	1.93 (0.87,8.15)	−2.20	0.0275
MCTSI	2 (0,2)	4 (2,5.5)	−5.3	<0.0001	2 (0,2)	4 (2,6)	−3.34	0.0008

In both the training set and validation set, the MCTSI scores in the progression group were higher than in the non-progression group (*Z* = –5.3; –3.34, *P* < 0.001).

### Radiomics analysis

**Figure 2:**
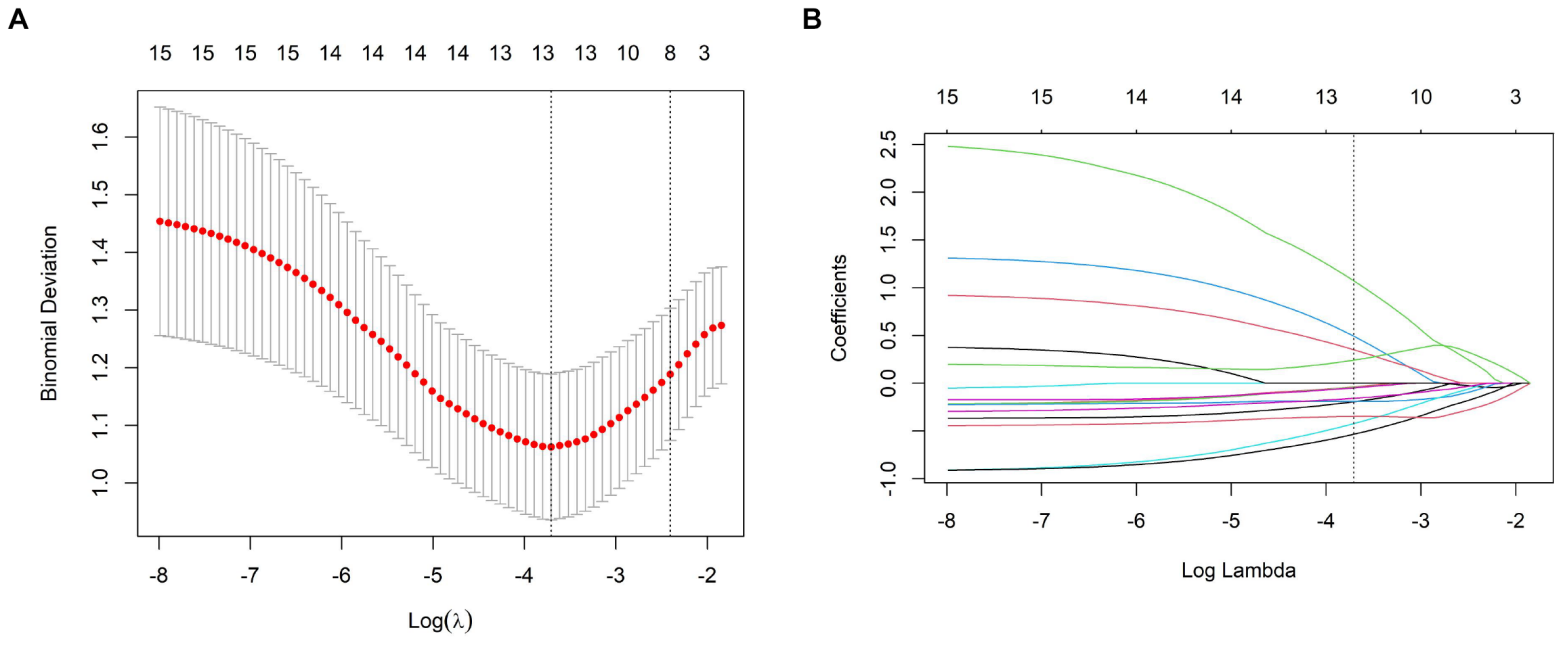
**(A)** The binomial deviation of the LASSO model in the training set samples with λ of the binomial deviation, and the binomial deviation with the smallest λ value as the best λ value. **(B)** Change of the coefficients of the variables in the LASSO model with λ.

**Figure 3:**
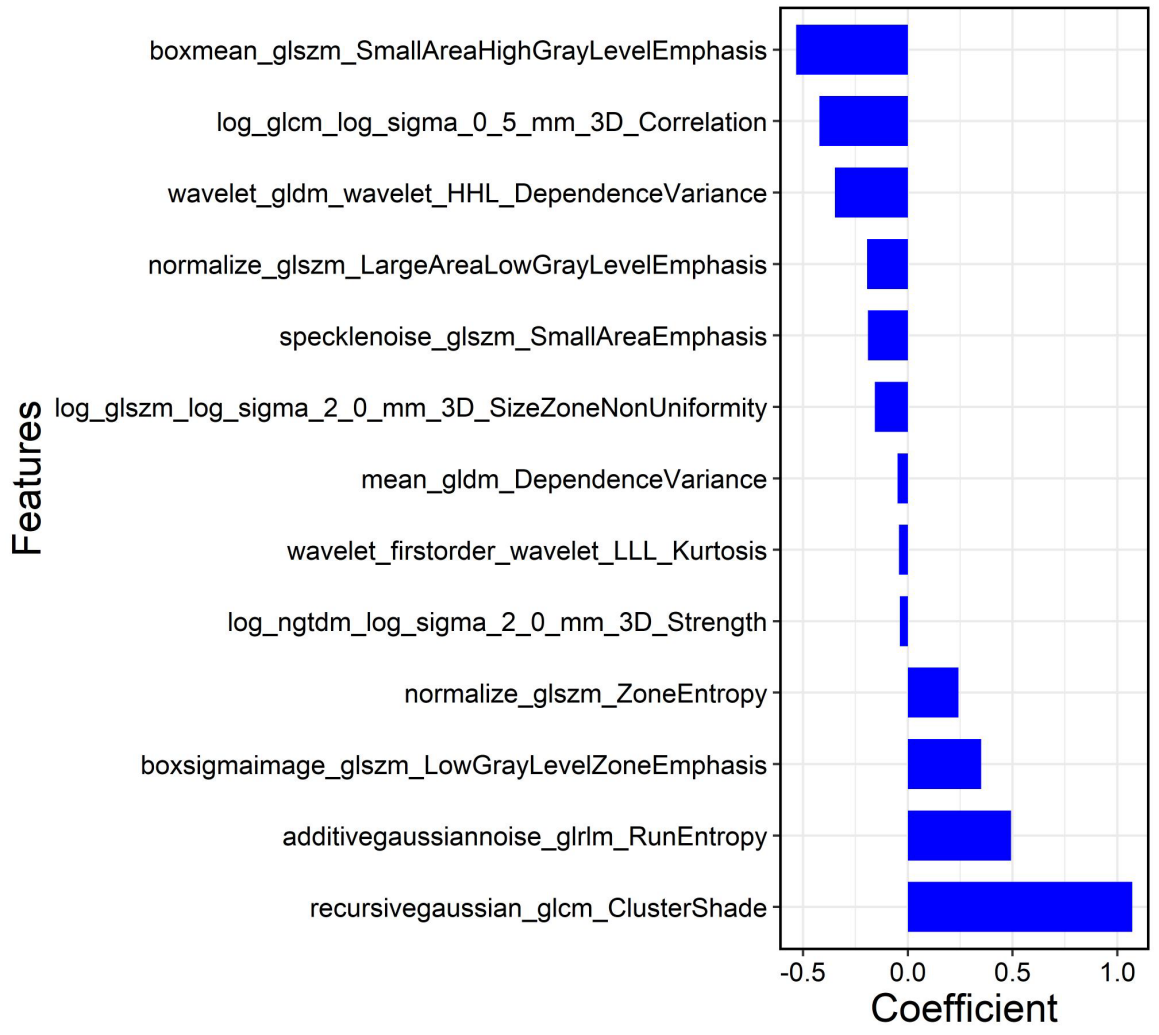
Coefficients of the 13 texture features selected by LASSO.

The meaningful texture features were obtained by extracting the texture and filtering the transform by the uAI Research Portal. Selection of the best parameters for binomial bias through *Z*-score, ICC, mRMR algorithm, and LASSO regression. Figure 2A shows that λ increases the variation of binomial deviation of the model on the training set samples. The value with the smallest binomial deviation is selected as the best parameter value. Figure 2B shows the variation of the coefficients of the variables in the model. Thirteen texture features with large values were selected from the CT plain scan images, and their corresponding coefficients are shown in Figure 3. Calculate the rad-score for each patient based on the following formula: Radscore = 1.0661 + 1.0718 × recursiveGaussian_glcm_ClusterShade+0.4923 × additivegaussiannoise_glrlm_RunEntropy+0.3503 × boxsigmaimage_glszm_LowGrayLevelZoneEmphasis+0.2422 × normalize_glszm_ZoneEntropy-0.0386 × log_ngtdm_log_sigma_2_0_mm_3D_Strength-0.0424 × wavelet_firstorder_wavelet_LLL_Kurtosis-0.048 × mean_gldm_DependenceVariance-0.1576 × log_glszm_log_sigma_2_0_mm_3D_SizeZoneNonUniformity-0.1904 × specklenoise_glszm_SmallAreaEmphasis-0.1940 × normalize_glszm_LargeAreaLowGrayLevelEmphasis-0.3478 × wavelet_gldm_wavelet_HHL_DependenceVariance-0.4221 × log_glcm_log_sigma_0_5_mm_3D_Correlation-0.5333 × boxmean_glszm_SmallAreaHighGrayLevelEmphasis.

The rad-score fused MCTSI is derived for each patient by calculating the coefficients of each texture feature to build a radiomics model.

**Figure 4:**
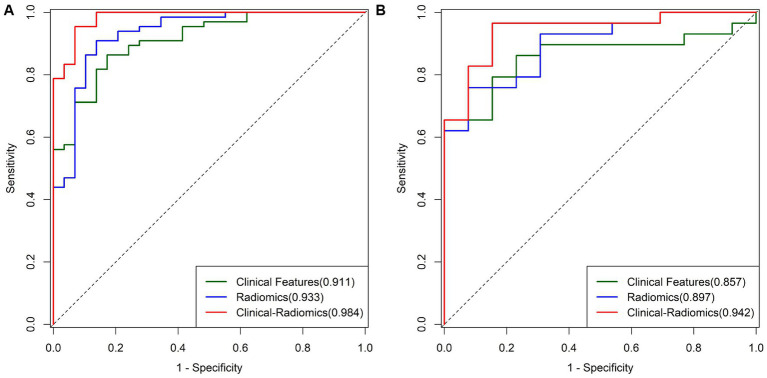
**(A)** ROC curves of different models in the training group. **(B)** ROC curves of different models in the validation group.

**Figure 5:**
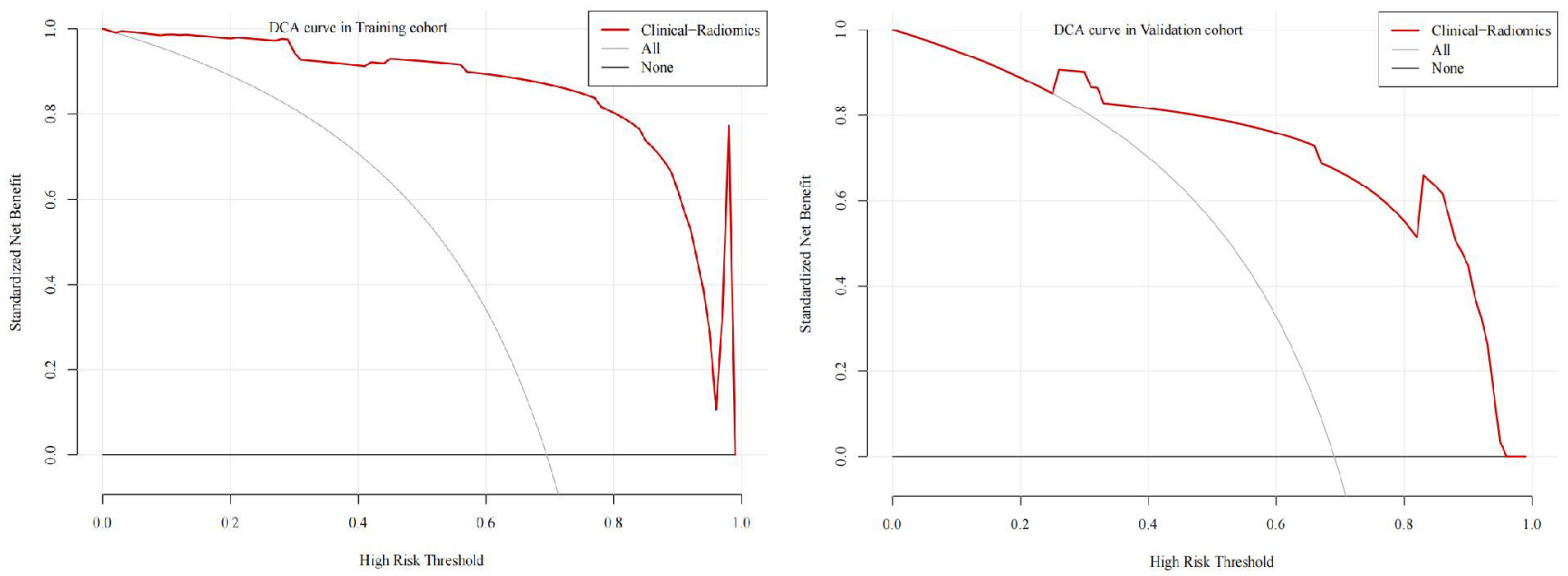
DCA of the models in the training set and validation set.

Based on the texture features of plain scan images, the optimal features are selected. By fusing MCTSI scores, establish clinical models, radiomics models, and clinical–radiomics models, respectively. According to the ROC curve (Table 4), the AUCs in the training set were 0.911, 0.933, and 0.984, respectively, and the AUCs in the validation set were 0.857, 0.897, and 0.942, respectively. In the training set, the clinical–radiomics model has the best predictive performance. Its predictive performance is higher than the clinical model (*Z* = 2.767, *p* = 0.005) and the radiomics model (*Z* = 2.033, *p* = 0.042) (Figure 4). There was no statistically significant difference in AUC between the training and validation sets for clinical models (*D* = 0.644, *p* = 0.522), radiomics models (*D* = 0.823, *p* = 0.414), and clinical–radiomics models (*D* = 1.108, *p* = 0.274). The results of DCA indicate that the clinical–radiomics model has a higher clinical net benefit (Figure 5).

**Table 4:** Clinical characteristics model, radiomics label, and radiomics prediction model ROC result.

Model	Training set (*n* = 95)						Validation set (*n* = 42)					
	AUC	ACC	Sensitivity	Specificity	PPV	NPV	AUC	ACC	Sensitivity	Specificity	PPV	NPV
Clinical features	0.911 (0.853–0.969)	0.853 (0.765–0.917)	0.864	0.828	0.919	0.727	0.857 (0.741–0.973)	0.81 (0.659–0.914)	0.793	0.846	0.920	0.647
Radiomics	0.933 (0.875–0.990)	0.895 (0.815–0.948)	0.909	0.862	0.938	0.806	0.897 (0.803–0.990)	0.81 (0.659–0.914)	0.759	0.923	0.957	0.632
Clinical–radiomics	0.984 (0.964–1.000)	0.947 (0.881–0.983)	0.955	0.931	0.969	0.900	0.942 (0.87–1.000)	0.929 (0.805–0.985)	0.966	0.846	0.933	0.917

### Assessment of clinical–radiomics features

The results of univariate and multivariate logistic regression analysis of clinical–radiomics features predicting the progression of acute pancreatitis are shown in Table 5. The results of univariate logistic regression analysis showed that BISAP, CRP, PCT, MCTSI, and rad-score were significant factors in differentiating the progression of acute pancreatitis. The results of multivariate logistic regression analysis showed that BISAP (OR = 6.187; 95% *CI:* 2.259–27.047; *p* = 0.003), PCT (OR = 1.923; 95% *CI*: 1.040–5.568; *p* = 0.124), and rad-score (OR = 3.841; 95% *CI*: 1.578–13.481; *p =* 0.013) were independent predictors of progression of acute pancreatitis, and two factors, MCTSI (OR = 1.281; 95% *CI:* 0.690–2.617; *p =* 0.455) and CRP (OR = 1.001; 95% *CI*: 0.992–1.010; *p =* 0.872), were not independent factors.

**Table 5:** Results of univariate and multivariate logistic regression analysis of clinical–radiomics characteristics.

Clinical–radiomics features	Univariate analysis		Multivariate analysis	
	OR (95% CI)	*P*-value	OR (95% CI)	*P*-value
BISAP	5.167 (2.630–12.094)	<0.001	6.187 (2.259–27.047)	0.003
CRP	1.006 (1.002–1.011)	0.014	1.001 (0.992–1.010)	0.872
PCT	2.034 (1.381–3.511)	0.003	1.923 (1.040–5.568)	0.124
MCTSI	2.658 (1.791–4.477)	<0.001	1.281 (0.690–2.617)	0.455
age	0.978 (0.949–1.007)	0.139		
gender	2.057 (0.819–5.558)	0.136		
Rad-score	5.037 (2.799–10.728)	<0.001	3.841 (1.578–13.481)	0.013

## Discussion

The initial diagnosis of AP patients accounts for approximately 0.3% of the total number of emergency department patients. The pathogenesis is due to the abnormal activation of pancreatic enzymes. The pancreatic enzyme causes damage to the pancreas itself and surrounding organs, which, in turn, triggers related inflammatory and immune responses. In severe cases, it can lead to organ dysfunction and even death (21). There is evidence that mild and acute pancreatitis is associated with hyperperfusion within the first few hours of symptom onset, while moderate and severe pancreatitis is accompanied by complications, and pancreatic parenchyma has progressive tissue ischemia and decreased perfusion. It has been suggested that damage to the pancreatic parenchyma from microcirculatory disorders is present at an early stage, and it is difficult to reflect such changes by conventional CT plain scan (22). In this study, we established and validated a clinical–radiomics model based on CT plain scan images to predict the progression of AP in a non-invasive and quantitative way.

Majidi et al. (23) found CRP and PCT in the SAP group were higher than in the MAP group (*p* < 0.05). With the progressing pancreatitis, PCT and CRP also increased. BISAP can early identify patients with a high risk of complications and death using five simple indicators (12). Singh et al. (22) found that the BISAP can effectively predict the severity of AP. They found that gender was balanced by comparing clinical baseline data. Khanna et al. (10) found that the age of AP patients is between 21 and 50 years old. By comparing the clinical characteristics of the training set and the validation set, we found that the BISAP, CRP, and PCT in the progressive group were higher than those in the non-progressive group, and the age in the progressive group was smaller than in the non-progressive group.

Balthazar et al. (24–26) established the CT Severity Index (CTSI). It grades and scores the degree of pancreatitis and necrosis to predict the incidence rate and mortality of AP. Later, Mortele et al. (27) improved its limitations by incorporating numerical scores for extrapancreatic complications and establishing MCTSI. Its correlation with clinical outcomes and local complications is superior to CTSI. Banday et al. (28) found that compared to CTSI, MCTSI is simpler and more accurate and has a stronger correlation with clinical outcomes, including hospital stay, infection development trends, organ failure, mortality rate, and the need for intervention treatment. Therefore, we fused MCTSI to the radiomics model to improve the predictive performance. Radiomics can reveal information hidden in conventional images to reflect potential biological and pathological changes.

In the early stage of a conventional CT plain scan, radiomics may reflect damage to the pancreatic parenchyma. In this study, a series of radiomics features of the first order, shape, glcm, glrlm, glszm, gldm, and ngtdm parameters were extracted with high throughput. They provided a comprehensive description of the morphology, radiographic attenuation, and texture information of the lesion. After the dimensionality reduction and radiomics analysis, the 13 most valuable texture features were selected. Then, the rad-score was calculated to obtain. The radiomics feature with the maximum absolute values in LASSO is recursivegaussian_glcm_ClusterShade. Cluster Shade is a measure of the skewness and uniformity of the GLCM. It is used to describe the joint distribution of two pixels with some spatial relationship. ClusterShade is associated with the heterogeneity of voxels in the area of interest. The maximum absolute values in LASSO indicate that the internal structure of the pancreatic parenchyma varies greatly due to inflammation or necrosis caused by leakage of pancreatic fluid. In addition, less first-order feature extraction may be related to the exudate parenchymal necrosis of inflammation resulting in poorer display of anatomical details. Moreover, we obtain the texture features containing wavelet filter decomposition and local binomial transform account for more. The image signal can be decomposed into subbands. Using different algorithms for different subbands aims to highlight approximation and details at different scales. Wavelet transforms have important image analysis capabilities. The wavelet transform coefficients can provide us with edge information of the lesion for extracting relevant radiomics features. The local binomial transform can extract the original feature information and spatial positional relationships of the image pixels. This analysis method has the advantages of rotation and gray-scale invariance (29). It can effectively reflect the invariance of local information in a CT plain scan (30).

Therefore, we establish a radiomics model with rad-score and MCTSI. We use clinical baseline data to establish a single predictive model. By comparing the AUC, we found that the performances of the radiomics model and the clinical model were good and stable. Finally, the radiomics model was fused with clinical baseline data to obtain a joint clinical–radiomics model. The performance has been further improved, with statistically significant differences compared to single clinical models or radiomics models. Moreover, the diagnostic efficiency of the clinical–radiomics model is the highest in both the training and validation sets. Moreover, there is no statistical difference between the two sets, which shows that the model obtained through further research has the best and stable predictive performance. Early prediction of patient progression can be achieved through quantitative analysis of several simple clinical data and patient CT plain scan images.

### Limitations of this study

(1) The analysis of this study was based on radiomics features extracted from two-dimensional images of the pancreatic parenchyma at the largest level of the diseased pancreas, and it may be more effective to reflect the lesions if three-dimensional images of the entire pancreatic parenchyma are extracted; (2) a retrospective single-center study was conducted with hospitalized patients. There may be significant bias in sample selection; (3) AP patients in our hospital undergo routine CT plain scans upon admission. If conducting radiomics analysis based on CECT scanning images, a more efficient model can be obtained; (4) patients with mild symptoms do not need image examinations to diagnose and manage AP. Currently, there are few imaging studies on patients with mild symptoms. Moreover, our results tend to analyze more severe and complicated pancreatitis.

In summary, the clinical–radiomics model based on pancreatic parenchymal CT has good predictive performance in both the training and validation sets, helping to identify and judge the progression trend of acute pancreatitis patients as soon as possible, and taking timely and effective measures to improve the patient’s prognosis.

## Data availability statement

The raw data supporting the conclusions of this article will be made available by the authors, without undue reservation.

## Ethics statement

The studies involving humans were approved by the Ethics Committee of First Affiliated Hospital of Harbin Medical University. The studies were conducted in accordance with the local legislation and institutional requirements. The ethics committee/institutional review board waived the requirement of written informed consent for participation from the participants or the participants’ legal guardians/next of kin because (1) this study only retrospectively analyzed the patient’s imaging and clinical data, without any intervention on the patient’s condition, and there is no risk to the life and health of the subjects. (2) For the collection or research of previously archived data, files, records, and imaging data, researchers are unable to contact the subjects.

## Author contributions

MX: Conceptualization, Methodology, Writing – original draft. SL: Data curation, Methodology, Writing – original draft. DX: Data curation, Writing – original draft. HW: Formal analysis, Resources, Writing – original draft. QG: Investigation, Writing – original draft. LZ: Investigation, Writing – original draft. XX: Conceptualization, Project administration, Writing – review & editing. YJ: Methodology, Writing – review & editing.
